# Sinus Rhythm Conduction Properties across Bachmann’s Bundle: Impact of Underlying Heart Disease and Atrial Fibrillation

**DOI:** 10.3390/jcm9061875

**Published:** 2020-06-16

**Authors:** Christophe P. Teuwen, Lisette J.M.E. van der Does, Charles Kik, Elisabeth M.J.P. Mouws, Eva A.H. Lanters, Paul Knops, Yannick J.H.J. Taverne, Ad J.J.C. Bogers, Natasja M.S. de Groot

**Affiliations:** 1 Department of Cardiology, Erasmus University Medical Center, Doctor Molewaterplein 40, 3015 GD Rotterdam, The Netherlands; c.teuwen@erasmusmc.nl (C.P.T.); j.vanderdoes@erasmusmc.nl (L.J.M.E.v.d.D.); e.mouws@erasmusmc.nl (E.M.J.P.M.); e.lanters@erasmusmc.nl (E.A.H.L.); p.knops@erasmusmc.nl (P.K.); 2 Department of Cardio-Thoracic Surgery, Erasmus University Medical Center, Doctor Molewaterplein 40, 3015 GD Rotterdam, The Netherlands; c.kik@erasmusmc.nl (C.K.); y.j.h.j.taverne@erasmusmc.nl (Y.J.H.J.T.); a.j.j.c.bogers@erasmusmc.nl (A.J.J.C.B.)

**Keywords:** epicardial mapping, sinus rhythm, atrial fibrillation, valvular heart disease, Bachmann’s Bundle

## Abstract

Valvular heart disease (VHD) is a common risk factor for atrial fibrillation (AF). Conduction abnormalities (CA) during sinus rhythm (SR) across Bachmann’s bundle (BB) are associated with AF development. The study goal is to compare electrophysiological characteristics across BB during SR between patients with ischemic (IHD) and/or VHD either with or without ischemic heart disease ((I)VHD), with/without AF history using high-resolution intraoperative epicardial mapping. In total, 304 patients (IHD: *n* = 193, (I)VHD: *n* = 111) were mapped; 40 patients (13%) had a history of AF. In 116 patients (38%) there was a mid-entry site with a trend towards more mid-entry sites in patients with (I)VHD vs. IHD (*p* = 0.061), whereas patients with AF had significant more mid-entry sites than without AF (*p* = 0.007). CA were present in 251 (95%) patients without AF compared to 39 (98%) with AF. The amount of CA was comparable in patients with IHD and (I)VHD (*p* > 0.05); AF history was positively associated with the amount of CA (*p* < 0.05). Receiver operating characteristic (ROC) curve showed 85.0% sensitivity and 86.4% specificity for cut-off values of CA lines of respectively ≤ 6 mm and ≥ 26 mm. Patients without a mid-entry site or long CA lines (≥ 12 mm) were unlikely to have AF (sensitivity 90%, *p* = 0.002). There are no significant differences in entry-sites of wavefronts and long lines of CA between patients with IHD compared to (I)VHD. However, patients with AF have more wavefronts entering in the middle of BB and a higher incidence of long CA lines compared to patients without a history of AF. Moreover, in case of absence of a mid-entry site or long line of CA, patients most likely have no history of AF.

## 1. Introduction

Propagation of electrical wavefronts during sinus rhythm (SR) occurs from the right atrium towards the left atrium through different connections such as the coronary sinus, fossa ovalis and Bachmann’s bundle (BB) [1]. Because of limited access to the epicardially located BB, electrical activation across BB has rarely been studied. In patients with ischemic heart disease (IHD), it was recently shown that although BB was thought to be of paramount importance for interatrial conduction from the right to left atrium during SR, it was also activated by SR wavefronts emerging in the middle and left site of the bundle [2]. In addition, patients with atrial fibrillation (AF) had a higher degree of conduction disorders across BB. This observation suggests a possible role of BB in development of AF which has also been proposed by other investigators [3,4].

The suggested role of BB in AF development was mainly based on subtle electrocardiogram (ECG) changes [5]. These ECG findings were associated with clinical outcomes such as stroke and AF (Bayés syndrome) [5]. Furthermore, pacing at BB instead of the usual right atrial appendage might be effective for prevention of AF paroxysms and progression to persistent AF, although studies showed conflicting results [6,7].

Valvular heart disease (VHD) is one of the major risk factors predisposing to development of AF [8]. Conduction across BB might be affected by VHD, as VHD and conduction disorders across BB are both correlated to development of AF. However the effect of underlying heart disease such as VHD on conduction across BB is so far unknown in humans, as detailed activation mapping of BB has only been described in patients with IHD. The aim of the present study was (1) to examine electrophysiological properties during SR including entry sites and conduction disorders across BB during SR, (2) to compare these properties between patients with ischemic and/or valvular heart disease and (3) to correlate these electrophysiological properties with the occurrence of previous AF episodes.

## 2. Methods

### 2.1. Study Population

A total of 304 patients of at least 18 years of age who underwent open chest cardiac surgery for coronary artery bypass graft and/or VHD (aortic or mitral valve) were included. Patients were classified into 2 groups; IHD and VHD. The group of IHD mainly consists of patients analyzed in our previous report (*n* = 185) [2]. The latter containing patients with solely VHD and VHD in combination with IHD. As VHD is considered a leading risk factor for development of AF and to maintain sufficient statistical power by comparing similar group sizes, these patients are initially categorized as one: (I)VHD. However, complete sub-analyses for entry-sites and conduction disorders for patients with IHD only, IVHD and VHD only are shown in Supplement 1. Echocardiographic examination was part of standard protocol prior to the surgical procedure, whereas other imaging techniques (e.g., magnetic resonance imaging (MRI) were not. Patients were excluded in case of paced atrial rhythm, Wolff–Parkinson–White syndrome, severe renal failure, previous open chest cardiac surgery, prior ablative therapy, hemodynamic instability (presence of assist devices, usage of inotropic) and prior radiation for chest malignancies.

This study is part of the prospective observational projects QUASAR and HALT & REVERSE which were both approved by the Medical Ethical Committee in the Erasmus Medical Center (MEC 2010-054 and MEC 2014-393) [9]. Written informed consent was provided by all patients prior to the surgical procedure. 

### 2.2. Mapping Procedure

**Figure 1:**
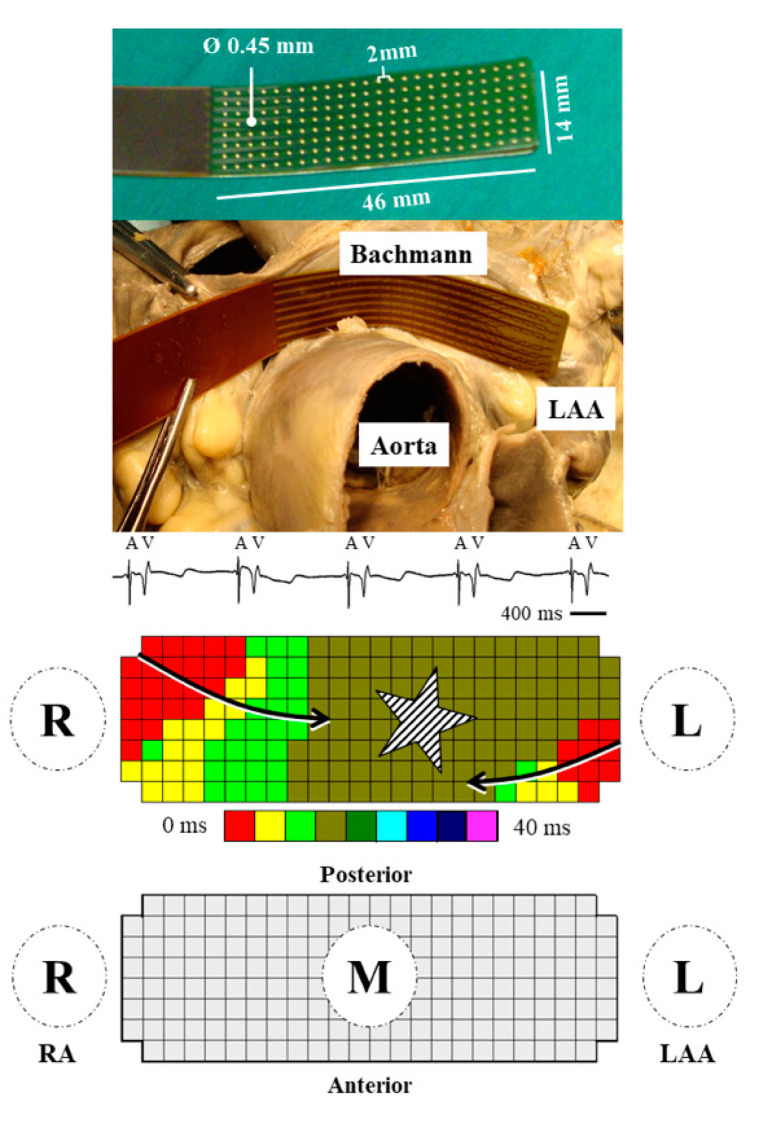
Mapping procedure of Bachmann’s bundle. (**Upper panel)** 192-unipolar electrode mapping array including measurements of length, inter-electrode distance and electrode diameter. The mapping array is subsequently positioned at Bachmann’s bundle, by placing the array behind the aorta with the tip against the left atrial appendage. (**Middle panel**) unipolar electrogram with steep atrial deflection (A) and far-field ventricular signal (V). After marking all atrial deflections, a color-coded activation map is constructed. The arrows depict direction of wavefront propagation. The striped star illustrates an area of simultaneous excitation/focal wave. In the current example, the mid-entry corresponds to location of the interatrial septum and transition of BB to left atrial roof (posterior). (**Lower panel**) Schematic overview of 192-unipolar electrode mapping array. Entry sites are denoted with R (right), M (middle) and L (left). IAS = interatrial septum; LAA = left atrial appendage; RA = right atrium.

High-resolution epicardial mapping was performed as previously described [2,9]. A bipolar pacemaker-wire was stitched to the right atrial free wall (terminal crest), serving as temporal reference electrode. A steal wire was fixed in the thoracic subcutaneous tissue serving as indifferent electrode. The initial 161 patients were mapped with a 128-unipolar electrode (8 × 16) mapping array, whereas the remaining patients were mapped with a mapping array containing 192-unipolar electrodes (8 × 24) (inter-electrode distance 2.0 mm) [2]. The mapping array was positioned on BB by placing it over the interatrial roof behind the aorta with the tip against the left atrial appendage (Figure 1, upper panel). Mapping of BB with the 128-electrode array was performed by shifting the array backwards towards the superior cavo-atrial junction resulting in 2 consecutive positions. Solely patients with electrical activation present at >75% of the mapping area were included. Although this may be the result of low voltage areas, limited contact of the mapping array on the myocardium cannot be excluded and therefore this cut-off value was chosen.

SR was recorded during 5 s, including a surface ECG lead, a calibration signal of 2 mV and 1000 ms, unipolar epicardial electrograms and a bipolar reference electrogram [2,9].

### 2.3. Mapping Data Analysis

Mapping data were analyzed using our custom-made software [2,9]. The steepest negative deflection of the unipolar atrial potentials was annotated as local activation time. Based on the activation times, color-coded activation maps were automatically constructed as demonstrated in the middle panel of Figure 1. An averaged beat was subsequently created after excluding premature and aberrant beats. The averaged maps were used for analysis of patterns of activation and quantification of conduction disorders. Patterns of activation were classified according to entry-sites; right, middle and left (Figure 1, lower panel). A wavefront entering the area under the mapping array from the right atrial side from where it propagates towards the left side was defined as right entry site, whereas in case this was observed vice versa it was defined as left entry site. An area of simultaneous excitation or a wavefront emerging in the center of the mapping array as focal wave was defined as mid-entry site [2]. In addition, wavefronts entering from the anterior or posterior borders in the middle part of the mapping array, were also defined as mid-entry. In a previous study from our group, we described the origin of these mid-entry wavefronts based on anatomy of dissected hearts. Either these wavefronts propagate from the interatrial septum upwards to BB, which are connected in some patients, or these wavefronts enter BB through parallel bundles that merge either on the anterior or posterior site of BB [10]. For quantification of conduction disorders, difference in local activation times between 2 adjacent electrodes were determined. Conform previous studies, conduction delay (CD) was determined as time differences of 7–11 ms (conduction velocity: <29 cm/s) between 2 adjacent electrodes. In case time difference was ≥12ms between 2 adjacent electrodes (conduction velocity: < 17cm/s), this area was marked as conduction block (CB) [2,9]. The amount of conduction delay and/or block was measured as a percentage of all inter-electrode conduction times. The number of lines of CD/CB and their length were measured separately. When lines of CD and CB were connected to each other, they were denoted as CDCB.

### 2.4. Statistical Analysis

Normally distributed data are described by mean ± SD, whereas skewed data are described by median (interquartile range) and categorical data as numbers and percentages. Normally distributed data are analyzed with Student’s T-test or one way ANOVA, skewed data with Kruskall–Wallis test or Mann–Whitney U-test and categorical data with χ^2^ or Fisher exact test when appropriate. The correlation between patient characteristics in the entire study population or IHD/(I)VHD separately and conduction disorders was performed using Spearman rank correlation. A correlation of 0.1–0.3 was considered weak, 0.3–0.5 moderate and >0.5 strong. For further clinical interpretation of observed conduction disorders, receiver operating characteristic curves (ROC-curves) from previous AF episodes were extracted to calculate CB/CDCB cut-off values for sensitivity and specificity. Subsequently, based on previous data showing an association between lines of CB ≥12 mm with development of postoperative AF, we also studied the relation of previous AF episodes and lines of CB/CDCB ≥12 mm. With current findings, we added a mid-entry site to these analysis and determined sensitivity and specificity with χ^2^. In addition, we investigated the relation between late postoperative AF (LPAF) using previous long-term follow-up data and presence of lines of CB/CDCB ≥12 mm and/or mid-entry site. LPAF was detected on ECG or 24-h Holter recordings, performed either on regular base or on indication during outpatient clinic control. Finally, the association of potential determinants (e.g., age, left ventricular function, left atrial dilatation) with CB/CDCB ≥12 mm or mid-entry site was tested using univariate binary logistic regression models. Due to skewness, age was transformed to a binary value with top 25% (≥72.5 years) set as ‘high’. Aging, gender and underlying heart disease were subsequently selected for multivariate binary logistic regression, next to determinants with a *p*-value ≤ 0.20. A *p*-value < 0.05 was considered statistically significant. Statistical Package of Social Sciences version 21.0 for Windows (SPSS Inc. Chicago, IL, USA) was used.

## 3. Results

### 3.1. Study Population

Study population characteristics (*n* = 304, 237 male (78%), age 66 ± 10 years) are shown in Table 1. The mean age in the entire study population was 66 ± 10 years. Patients had either IHD (*n* = 193, 63.5%), VHD (*n* = 62, 20.4%) or a combination of ischemic and valvular heart disease (*n* = 49, 16.1%). Patients underwent cardiac surgery different valvular pathology including aortic valve stenosis (*n* = 70, 23.0%), aortic valve insufficiency (*n* = 20, 6.6%), mitral valve stenosis (*n* = 3, 1.0%) and mitral valve insufficiency (*n* = 41, 13.5%). Two-hundred thirty patients (75.7) used anti-arrhythmic drugs prior to surgical procedure; Class II (*n* = 211, 69.4%), Class III (*n* = 9, 3.0%) and Class IV (*n* = 10, 3.3%). The majority of patients had a normal left ventricular function (*n* = 234, 77%) and only 10 patients (3%) had a moderate/severe left ventricular dysfunction. Left atrial dilatation was present in 54 patients (18%); half of them had isolated IHD.

A total of 40 patients (13%) had a history of AF; 32 paroxysmal, 7 persistent and 1 longstanding persistent. Of the latter two groups, all patients underwent electrical cardioversion prior to epicardial mapping. Comparing the presence of AF for different underlying heart disease, relatively most patients had AF in combination with mitral valve disease (*n* = 14, 34%), aortic valve disease (*n* = 12, 17%) and finally IHD solely (*n* = 14, 7%). Due to a limited number of patients with (longstanding) persistent AF, further comparison is not performed between different types of AF. Mapping was performed with mean rate of 72 ± 14 beats/min.

For further comparison of groups, patients were divided in having IHD or (I)VHD. The right side in Table 1 demonstrates differences between these groups. Although age was comparable (65.5 ± 9.2 vs. 66.8 ± 11.4), other characteristics which may potentially affect atrial conduction were different either with a higher incidence in patients with IHD including hypertension, hypercholesterolemia, diabetes mellitus, anti-arrhythmic drug usage and history of myocardial infarction (*p* ≤ 0.004) or a higher incidence in patients with (I)VHD such as left atrial dilatation and a history of AF (*p* ≤ 0.001).

### 3.2. Impact of Heart Disease and Atrial Fibrillation on Entry Sites

**Figure 2:**
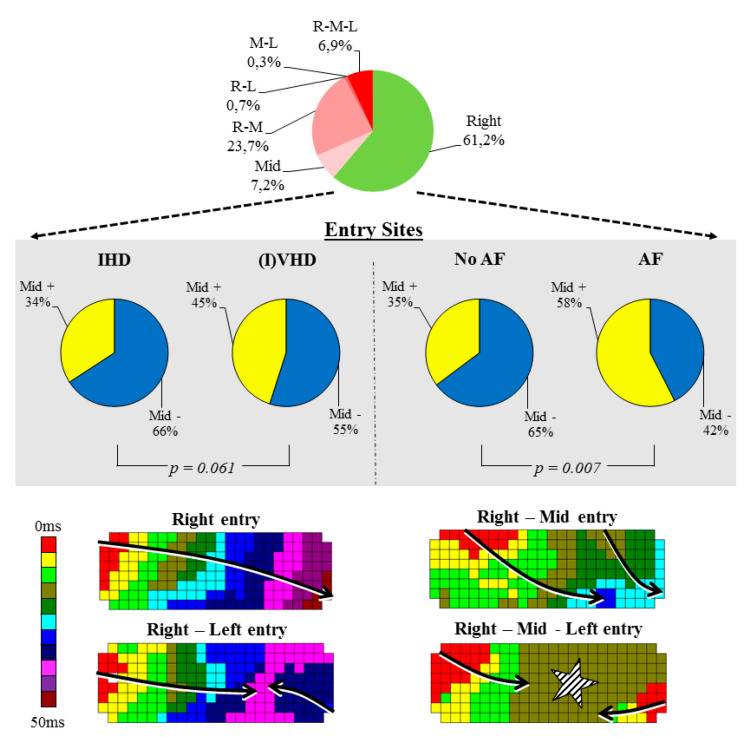
Entry sites and patterns of activation at Bachmann’s bundle. (**Upper panel**) Frequency pie illustrating all different entry sites in the entire study population including right entry site only (green) and other entry sites (red). (**Middle panel**) Frequency pies demonstrating the number of patients without a mid-entry site (blue) and with a mid-entry site (yellow) of wavefronts. The left panels illustrate the difference for underlying heart disease, the right panels for patients with/without a history of AF. (**Lower panel**) Examples of color-coded activation maps of BB during SR demonstrating different activation patterns; entry site only from the right (left upper map), right and middle (right upper map), right and left (left lower map) and right, middle and left (right lower map). Arrows indicate the main propagation direction of wavefronts, stars an area of simultaneous excitation/focal wave. AF = atrial fibrillation; IHD = ischemic heart disease; (I)VHD = (ischemic) valvular heart disease; L = left entry; M = mid entry; R = right entry.

We investigated whether the underlying heart disease and/or a history of AF has a relation with the number of wavefront entry sites into BB during SR and the location of these entry sites (right, middle, left or combinations). In total, the number of entry sites was either 1 site solely in 211 patients (69%) or multiple sites (2 sites: *n* = 73, 24%, 3 sites: *n* = 20, 7%). As BB is a major route of interatrial conduction, the vast majority of patients (*n* = 23, 92%) had at least 1 wavefront entering BB from only the right (*n* = 186, 61%) or a right entry site combined with other entry sites (*n* = 95, 31%) (Figure 2, upper panel). Furthermore, 116 patients (38%) had a wavefront entering BB in the middle including an entry site in the middle only (*n* = 22, 7.2%), right and middle (*n* = 72, 23.7%), middle and left (*n* = 1, 0.3%) and right, middle and left (*n* = 21, 6.9%).

Whereas the number of entry sites was comparable between patients with IHD (*n* = 59, 31%) and (I)VHD (*n* = 37, 33%, *p* = 0.48), patients with AF had more often >1 entry-site than patients without a history of AF (*n* = 19, 48% vs. *n* = 77, 29%; *p* = 0.02). Additionally, the middle panel of Figure 2 demonstrates that patients with a history of AF had more frequently a wavefront entering in the middle of BB compared to patients without AF (*n* = 23, 58% vs. *n* = 93, 35%; *p* = 0.007). In comparison, there was only a trend towards a higher incidence of mid entry sites in patients with (I)VHD compared to IHD (*p* = 0.061). For all 3 groups separately, solely patients with IVHD combined with AF had more mid-entry sites, although this group only consisted of 6 patients (see Table S1).

### 3.3. Correlation between Heart Disease or Atrial Fibrillation with Conduction Disorders

**Figure 3:**
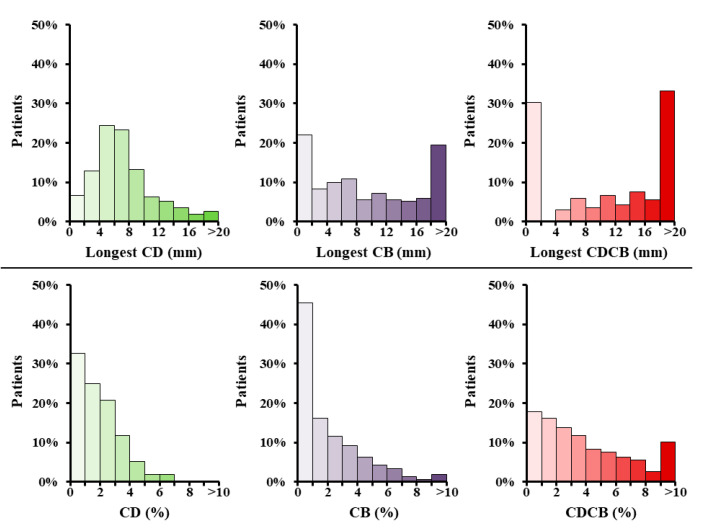
Incidence and extensiveness of conduction disorders. (**Upper panels**) Frequency histograms depicting the longest measured line of conduction delay (green), block (purple) and connected conduction delay and block (red) per patient. (**Lower panels**) Frequency histogram illustrating the percentage of conduction delay (green), block (purple) and combined (red) per patient. CB = conduction block; CD = conduction delay; CDCB (mm) = length of connected conduction delay and block; CDCB (%) = sum of conduction delay and block.

A total of 283 (93%) patients had at least 1 area of CD, 236 (78%) patients CB and 212 (70%) patients a continuous line of CDCB. In these patients, the longest lines of CD, CB and CDCB consisted of respectively 6 mm (4–8), 6 mm (2–16) and 12 mm (0–22) (Figure 3, upper panels). 

In the entire study population, a median of 1.8% (0.9–2.9) CD, 1.2% (0.3–3.2) CB and 3.2% (1.6–6.0) continuous lines of CDCB was measured, as demonstrated in the lower panels Figure 3). Although there was a significant positive correlation between the amount of CDCB and aging in the entire study population, the correlation was solely moderate (rho correlation 0.326, *p* < 0.001). Furthermore, in patients with (I)VHD, diabetes mellitus and left atrial dilatation was weakly correlated with the amount of CDCB, respectively rho 0.257 (*p* = 0.007) and rho 0.282 (*p* = 0.008), whereas the remaining patient characteristics demonstrated no correlation. Furthermore, the amount of conduction disorders is comparable between patients with IHD only, IVHD and VHD only (Table S1).

**Figure 4:**
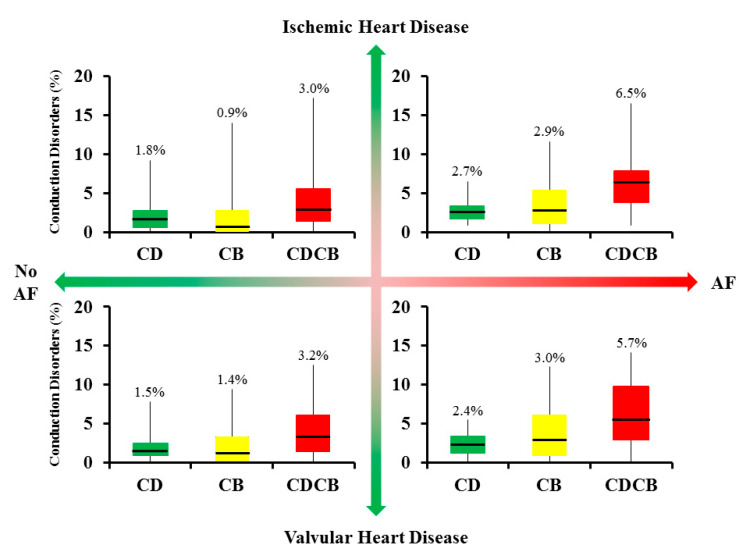
Relation between underlying heart disease, atrial fibrillation and conduction disorders Differences in the amount of conduction delay (green), block (yellow) and combined (red) between patients with ischemic heart disease (**upper panels**) and valvular heart disease (**lower panels**). In addition, difference in conduction disorders are shown between patients without atrial fibrillation (**left panels**) and with a history of atrial fibrillation (**right panels**). AF = atrial fibrillation; CB = conduction block; CD = conduction delay; CDCB = sum of conduction delay and block.

Figure 4 demonstrates conduction disorders in patients with IHD (upper panels), (I)VHD (lower panels), without a history of AF (left panels) and with a history of AF (right panels). As shown in Figure 4, the amount of conduction disorders is nearly comparable between patients with IHD and (I)VHD; CB 0.9% vs. 1.4% (*p* = 0.155) and CDCB 3.0 vs. 3.2% (*p* = 0.488) in patients without a history of AF. Additionally, in patients with a history of AF there were no significant differences between IHD and (I)VHD; CB 2.9% vs. 3.0% (*p* = 0.90) and CDCB 6.5% vs. 5.7% (*p* = 0.79) (see also Table S1 for separate analyses).

In total, 39 (98%) patients with AF and 251 (95%) without AF had at least some areas of CDCB. However, patients with AF, both with IHD and (I)VHD, have a higher amount of CB and CDCB compared to patients without a history of AF, respectively IHD 0.9% vs. 2.9% CB (*p* = 0.019), 3.0% vs. 6.5% CDCB (*p* = 0.006) and (I)VHD 1.4% vs. 3.0% CB (*p* = 0.018) and 3.2 vs. 5.7% CDCB (*p* = 0.015).

In line with these results, patients with early postoperative AF also had a higher amount of conduction disorder, respectively IHD 0.9% vs. 1.7% CB (*p* = 0.022), 2.7% vs. 4.2% CDCB (*p* = 0.026) and (I)VHD 1.5% vs. 1.7% CB (*p* = 0.119) and 3.4 vs. 3.8% CDCB (*p* = 0.030).

Furthermore, long-term follow-up was present in 266 patients (88%) with a median follow-up period of 24 months (range 3–36). In these patients, solely 10 patients (4%) developed LPAF of whom 8 patients had pre-operative AF. Comparison between patients with/without LPAF and the amount of conduction disorders was not performed due to the limited number of patients with LPAF.

### 3.4. Diagnostic Value for Atrial Fibrillation

**Figure 5:**
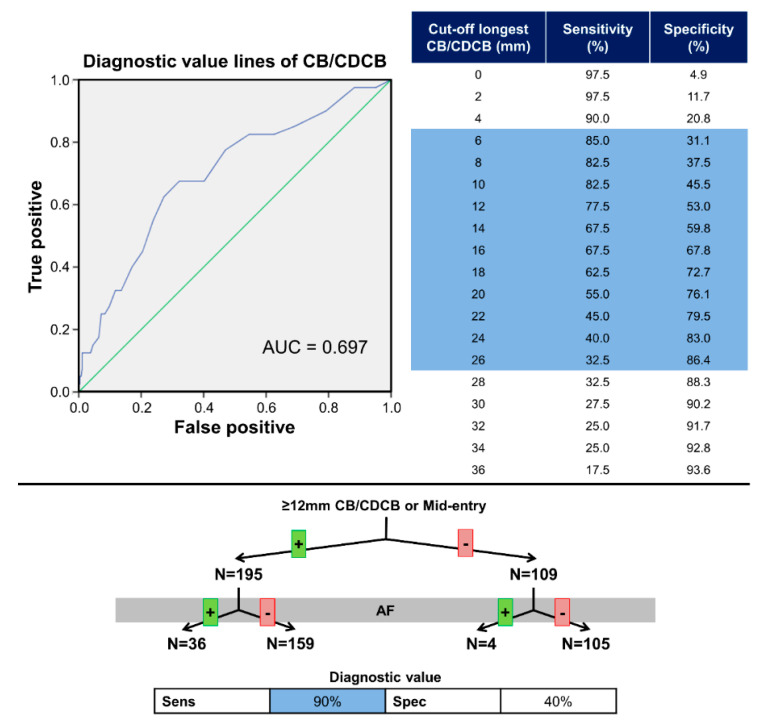
Predictive value of entry-site and conduction disorders. (**Upper panels**) Predictive value of the length of conduction disorders for previous AF episodes. The left panel depicts a ROC curve for length of lines of conduction disorders, The right panel cut-off values of the length of conduction disorders and previous AF episodes. (**Lower panel**) Flowchart demonstrating the predictive value of mid-entry site and a line of conduction block or CDCB of 12mm or more. Table shows sensitivity and specificity. AF = atrial fibrillation; CB = conduction block; CDCB = connected conduction delay and block; Sens = sensitivity; Spec = specificity.

Figure 5 illustrates the diagnostic value of longest CB/CDCB for AF. The diagnostic value of the longest lines of CB/CDCB is shown in the ROC-curve in Figure 5 with an area under the curve of 0.697. In addition, cut-off values for high sensitivity and specificity (≥85%) are respectively 6 mm and 26 mm (Figure 5, right upper panel).

The diagnostic value of a mid-entry and previous AF episodes was studied. As mentioned, patients with AF had relatively more frequently a wavefront entering in the middle of BB (see also Table S1). A total of 116 patients (38%) had a mid-entry of whom 23 patients (58%) had AF, leading to a sensitivity and specificity of respectively 58% and 65%. In addition, patients with AF, as previously described, had more conduction disorders. Thirty patients (75%) with AF and 124 patients (47%) without AF had a line of CB or CDCB ≥12 mm, resulting in a sensitivity of 75% and specificity of 53% for previous episodes of AF.

When combining these results, a mid-entry or a line of CB/CDCB ≥12 mm, nearly all patients with AF (*n* = 36, 90%) met these criteria compared to 159 patients (60%) of patients without AF (Figure 5, lower panel). Therefore, although there is a significant group of patients without AF with a mid-entry or CB/CDCB ≥12 mm, a patient was highly unlikely to have AF in the absence of these criteria (sensitivity 90%). Absence of one of these electrophysiological criteria is strongly associated with patients without AF (*p* = 0.002). If both a mid-entry and a line of CB/CDCB ≥12 mm are present, sensitivity is reduced to 50%. Furthermore, selection of patient characteristics (*p* ≤ 0.20 univariate analysis) for multivariate analysis including age, gender, history of AF, IHD/(I)VHD and diabetes mellitus demonstrated a history of AF (*p* = 0.007) and aging (*p* = 0.009) were both significantly associated with a mid-entry or CB/CDCB ≥12 mm, whereas gender (*p* = 0.72), (I)VHD (*p* = 0.32) and diabetes mellitus (*p* = 0.16) were not.

## 4. Discussion

The current study demonstrates that both patients with IHD and (I)VHD mainly have propagation of SR wavefronts across BB from the right towards the left atrial appendage. However, in over one third of patients, a wavefront emerges in the middle of BB towards surrounding sites. Furthermore, nearly all patients have conduction disorders across BB. There are no significant differences in wavefronts emerging in the middle of BB or the amount of conduction disorders between patients with IHD and (I)VHD. In contrast, patients with previous episodes of AF have more conduction disorders and more frequently a wavefront entering BB in the middle compared to patients without a history of AF. Taking both electrophysiological properties into account, patients without a mid-entry site or long lines of conduction disorders seldom have AF.

### 4.1. Atrial Remodeling in Atrial Fibrillation

Both cardiovascular and non-cardiovascular diseases contribute to development of AF. However, how these different diseases exactly contribute to AF development is still not completely unraveled. In general, several mechanisms have been proposed to underlie AF, including an ectopic rapid firing focus or reentry from which waves originate with fibrillatory conduction or conduction of multiple wavelets [11]. Moreover, electrical asynchrony between the epi- and endo-cardial layers was recently found as potential cause for maintenance of AF [12]. Irrespective of the underlying mechanism, conduction abnormalities (e.g., due to atrial fibrosis) have always been found to increase AF vulnerability. Müller-Edenborn et al. performed endocardial high-density voltage mapping in patients with persistent AF undergoing pulmonary vein isolation [13]. They investigated the location of areas or bipolar low voltages in the left atrium (peak-to-peak <0.5 and <1.0 mV) during SR. Subsequently, they related these areas of low voltages with alterations of P-wave morphology and risk of AF recurrence. The investigators concluded that low voltage areas are most often observed at the anteroseptal left atrial area which consists of myocardial fibers originating from BB. Furthermore, depending on location of low voltages in the left atrium, P-wave morphology may be altered and increased. Moreover, P-wave alterations and areas of low voltage enable risk stratification of AF recurrence after pulmonary vein isolation. They suggested, also based on previous anatomical studies [14], that conduction disorders might be correlated to local fibrosis. In our previous study focusing on conduction across BB in patients with IHD, we observed that patients with AF have a higher amount and longer lines of conduction disorders across BB compared to patients without AF [2]. As expected, the current study illustrates again that patients with AF have more and longer lines of conduction disorders. In line with findings by Müller-Edenborn et al., conduction disorders observed in the current study may be the result of local fibrosis. Although conduction disorders at BB may reflect pathology through the entire atrial myocardium such as the anteroseptal insertion, in our preliminary data with epicardial mapping of the entire atrial surface, conduction disorders seem mainly limited to BB in patients with AF which was not shown in the current study due to the extensiveness of data [15]. However, it remains unknown whether conduction disorders at BB facilitated development of AF or whether AF episodes further increased the amount of conduction disorders.

It is commonly known that atrial remodeling during AF enhances AF maintenance (“AF begets AF”) [16]. AF initiates electrical remodeling and is considered a cause of progression to persistent AF. In brief, electrical remodeling consists of, e.g., shortening of atrial refractoriness due to ion-channels adaptations [17,18,19,20]. The remodeling is reversible; time until normal state depends on the duration of AF. Next to electrical remodeling during AF, structural remodeling has been characterized as well, such as myocyte hypertrophy, myolysis and accumulation of glycogen (dedifferentiation) [17,18,19,20]. It is still a matter of debate whether AF itself also causes degeneration of myocytes with fibrotic deposition. In the goat model of persistent AF, structural remodeling was observed without production of fibrosis after >20 weeks of persistent AF induced by rapid atrial pacing [18]. In contrast, others suggest that atrial fibrosis might be enhanced during AF which in turn makes AF more persistent and therapeutic resistant [19,20].

The current study showed that conduction disorders are more present in patients with previous AF episodes, but the cause of the higher amount of conduction disorders is unknown. This is a non-longitudinal observational study and therefore the previous effects of conditions such as hypertension (blood pressure alterations) and atrial pressure that change over time and which may contribute to conduction disorders remain poorly understood. In addition, we did not observe clear differences in conduction disorders between patients with IHD and (I)VHD after correction for AF history, although the incidence of AF was higher in patients with (I)VHD conform previous many clinical studies. The similar amount of conduction disorders between IHD and (I)VHD may be caused by the complex pathophysiology in patients with IHD (e.g., atrial ischemia, elevated left ventricular pressure, diastolic dysfunction) and VHD (e.g., myocyte loss, increased effective refractory period due to reversible interstitial fibrosis, diastolic atrial dilatation) [21,22]. Moreover, there were differences in patient characteristics such as gender, hypertension and diabetes mellitus that may have a confounding effect on conduction disorders. However, further analyses demonstrated either no significant effect or a weak significant correlation (rho < 0.30) in each group.

Altogether, this leads to a chicken-and-egg situation; does VHD contribute to conduction disorders across BB predisposing to AF development? Or does AF enhance production of fibrosis resulting in a higher amount of conduction disorders across BB? Future longitudinal and experimental studies could provide more insights in these unanswered questions.

### 4.2. Relation between Mid-Entry and Patients with Atrial Fibrillation

BB is described as an important inter-atrial connection for conduction of electrical wavefronts [5]. As expected, BB was in the majority of our patients activated from the right to left. However, in line with a previous study [2], we also observed SR wavefronts entering in the middle of BB. This pattern of activation was more frequently observed in patients with AF.

There are 2 possible explanations why patients with AF have a higher incidence of wavefronts activating BB from the middle area. First, patients with AF have significantly more conduction disorders across BB which are also frequently longer than in patients without AF. Due to these long lines of conduction disorders, wavefronts are forced to propagate outside BB and around these lines, subsequently entering BB in the middle (‘quasi mid-entry’) behind these lines of conduction disorders. Second, previously it was demonstrated that the interatrial septum has connections with BB that provides the possibility for wavefronts to propagate to the middle of BB [23]. Propagation of SR wavefronts across BB from either right to left or from the middle (septum) to surrounding areas could depend on 2 factors: distance (S) or conduction velocity (CV) from sinus node to BB. Dobrzynski et al. and Ho et al. previously described that the sinoatrial node is more a sleeve rather than a node like structure at the intercaval region [24,25]. In patients with AF, the sinus node origin may vary, resulting in a longer distance between the initial excitation site and the right side of BB (↑ S), although Li et al. did not always find a relation between origin of the sinus node (intranodal) ‘pacing’ area and earliest atrial activation sites [26]. Furthermore, patients with AF have more conduction disorders across BB. These conduction disorders might also be more present between the sinus node and BB such as the preferential upper sinoatrial conduction pathway [26]. As a result, wavefronts propagate slower towards the right side of BB (↓ CV) and, therefore, propagation occurs through a different faster route such as towards the septum and subsequently upwards to BB.

### 4.3. Study Limitations

High-resolution epicardial mapping was performed of BB, but conduction properties of the remainder of the atria were not described. Therefore, it is unknown what the effect of conduction disorders in the remaining of the atria is on for example wavefront entry sites. Simultaneous endo- and epicardial of the entire atria could provide more insight in e.g., wavefront propagation, but this is so far technically impossible. Furthermore, patients were using antiarrhythmic drugs including mainly Class II. Although some patients were using Class III antiarrhythmic drugs which may affect conduction properties, these effects were not analyzed due to the limited number of patients. Patients with AF episodes were included. However, asymptomatic AF episodes in patients might have been missed which could result in an underestimation of the number of patients with a history of AF. In line with that, both sensitivity and specificity of a mid-entry site and long lines of conduction disorders for the presence of AF episodes could be positively/negatively affected in case none of the AF episodes were missed. Moreover, this also accounts for LPAF as episodes of LPAF may have been missed during follow-up which made further analyses impossible, for e.g., conduction disorders and development of LPAF.

## 5. Conclusions

Conduction disorders are equally present between patients with IHD and (I)VHD, but patients with AF have more and longer lines of conduction disorders. Propagation of wavefronts across BB during SR occurs mainly from the right atrial site towards left atrial site, but wavefronts also emerge in the middle of BB. Wavefronts entering BB in the middle were seen in patients with all different types of underlying heart diseases, but these were especially observed in patients with a history of AF. Altogether, a wavefront entering BB in the middle and/or long lines of conduction disorders are associated with absence of previous AF episodes.

## Figures and Tables

**Table 1:** Patient characteristics.

	Total	IHD	(I)VHD	*p*-Value
Patients, n	304	193	111	
Age, years (mean ± SD)	66.0 ± 10.1	65.5 ± 9.2	66.8 ± 11.4	0.415
Male gender, *n* (%)	237 (78.0)	163 (84.5)	74 (66.7)	<0.001
BSA, m^2^ (mean ± SD)	2.02 ± 0.21	2.05 ± 0.20	1.96 ± 0.21	0.564
Hypertension, *n* (%)	170 (55.9)	120 (62.2)	50 (45.0)	0.004
Hypercholesterolemia, *n* (%)	111 (36.5)	84 (43.5)	27 (24.3)	0.001
Diabetes mellitus, *n* (%)	85 (28.0)	68 (35.2)	17 (15.3)	<0.001
AAD, *n* (%)	230 (75.7)	166 (86.0)	64 (57.7)	<0.001
PCI, *n* (%)	70 (23.0)	58 (30.1)	12 (10.8)	<0.001
Myocardial infarction, *n* (%)	94 (30.9)	85 (44.0)	9 (8.1)	<0.001
Indication VHD, *n* (%)				
VHD	62 (20.4)		62 (55.9)	
IVHD	49 (16.1)		49 (44.1)	
Aortic valve stenosis	70 (23.0)		70 (63.1)	
Aortic valve insufficiency	20 (6.6)		20 (18.0)	
Mitral valve disease	3 (1.0)		3 (2.7)	
Mitral valve insufficiency	41 (13.5)		41 (36.9)	
Left ventricular function				0.618
Normal	234 (77.0)	146 (75.6)	88 (79.3)	
Mild dysfunction	60 (19.7)	39 (20.2)	21 (18.9)	
Moderate dysfunction	8 (2.6)	6 (3.1)	2 (1.8)	
Severe dysfunction	2 (0.7)	2 (1.0)	0	
Left atrial dilatation >45 mm, *n* (%)	54 (17.8)	27 (14.0)	27 (24.3)	0.001
History of AF, *n* (%)	40 (13.2)	14 (7.3)	26 (23.4)	<0.001
Paroxysmal	32 (10.5)	14 (7.3)	18 (16.2)	
Persistent	7 (2.3)	0	7 (6.3)	
Longstanding persistent	1 (0.3)	0	1 (0.9)	

AAD = antiarrhythmic drugs; AF = atrial fibrillation; BSA = body surface area; IHD = ischemic heart disease; (I)VHD = (ischemic) valvular heart disease; PCI = percutaneous coronary intervention; SD = standard deviation.

## Supplementary Materials

The following are available online at https://www.mdpi.com/2077-0383/9/6/1875/s1, Table S1: Sub-analyses electrophysiological characteristics in patients with ischemic heart disease only, valvular heart disease only and ischemic and valvular heart disease combined.
