# Supplementary material for: Sinus Rhythm Conduction Properties across Bachmann’s Bundle: Impact of Underlying Heart Disease and Atrial Fibrillation
Source: J Clin Med. 2020 Jun 16;9(6):1875. doi: 10.3390/jcm9061875 (PMC7355925; doi:10.3390/jcm9061875)
Supplement: Supplementary file 1 [file jcm-09-01875-s001.pdf]

Journal of
Clinical Medicine

MDPI

Table S1. Sub-analyses electrophysiological characteristics in patients with ischemic heart disease only, valvular heart disease only and ischemic and valvular heart disease combined.

	IHD	VHD	IVHD	
**CD (%), med (Q)**	1.8 (0.9 – 2.9)	1.6 (0.9 – 2.7)	1.8 (0.9 – 3.0)	ns
**CB (%), med (Q)**	1.1 (0 – 2.9)	1.3 (0.2 – 2.9)	2.1 (0.7 – 4.5)	ns
**CDCB (%), med (Q)**	3.0 (1.5 – 5.6)	3.2 (1.5 – 5.5)	3.8 (2.3 – 7.3)	ns
Total, N	193	62	49	
**Mid-entry (%)**	66 (34%)	28 (45%)	22 (45%)	ns
**CDCB ≥12mm (%)**	90 (46%)	35 (56%)	29 (59%)	ns
**Mid-entry or CDCB ≥12mm (%)**	116 (60%)	44 (71%)	35 (71%)	ns
No AF, N	179	42	43	
**Mid-entry (%)**	60 (34%)	17 (40%)	16 (37%)	ns
**CDCB ≥12mm (%)**	81 (45%)	19 (45%)	24 (56%)	ns
**Mid-entry or CDCB ≥12mm (%)**	104 (58%)	26 (61%)	29 (67%)	ns
AF	14	20	6	
**Mid-entry (%)**	6 (43%)	11 (55%)	6 (100%)*	
**CDCB ≥12mm (%)**	9 (64%)	16 (80%)	5 (83%)	ns
**Mid-entry or CDCB ≥12mm (%)**	12 (86%)	18 (90%)	6 (100%)	ns

AF = atrial fibrillation; **CD** = conduction delay; **CB** = conduction block; **CDCB** = connected conduction delay and block; **IHD** = ischemic heart disease; **IVDH** = ischemic and valvular heart disease; **med** = median; **N** = number; **ns** = not significant ($p > 0.05$); **Q** = quartiles; **VHD** = valvular heart disease.
